# Human monoclonal antibodies against Ross River virus target epitopes within the E2 protein and protect against disease

**DOI:** 10.1371/journal.ppat.1008517

**Published:** 2020-05-04

**Authors:** Laura A. Powell, Julie M. Fox, Nurgun Kose, Arthur S. Kim, Mahsa Majedi, Robin Bombardi, Robert H. Carnahan, James C. Slaughter, Thomas E. Morrison, Michael S. Diamond, James. E. Crowe

**Affiliations:** 1 Department of Pathology, Microbiology and Immunology, Vanderbilt University Medical Center, Nashville, Tennessee, United States of America; 2 Department of Medicine, Washington University School of Medicine, Saint Louis, Missouri, United States of America; 3 Vanderbilt Vaccine Center, Department of Pediatrics, Nashville, Tennessee, United States of America; 4 Department of Pathology and Immunology, Washington University School of Medicine, Saint Louis, Missouri, United States of America; 5 Department of Pediatrics, Vanderbilt University Medical Center, Nashville, Tennessee, United States of America; 6 Department of Biostatistics, Vanderbilt University, Nashville, Tennessee, United States of America; 7 Department of Immunology and Microbiology, University of Colorado, Aurora, Colorado, United States of America; 8 Department of Molecular Microbiology, Washington University School of Medicine, Saint Louis, Missouri, United States of America; 9 Andrew M. and Jane M. Bursky Center for Human Immunology and Immunotherapy Programs, Washington University School of Medicine, Saint Louis, Missouri, United States of America; Emory University, UNITED STATES

## Abstract

Ross River fever is a mosquito-transmitted viral disease that is endemic to Australia and the surrounding Pacific Islands. Ross River virus (RRV) belongs to the arthritogenic group of alphaviruses, which largely cause disease characterized by debilitating polyarthritis, rash, and fever. There is no specific treatment or licensed vaccine available, and the mechanisms of protective humoral immunity in humans are poorly understood. Here, we describe naturally occurring human mAbs specific to RRV, isolated from subjects with a prior natural infection. These mAbs potently neutralize RRV infectivity in cell culture and block infection through multiple mechanisms, including prevention of viral attachment, entry, and fusion. Some of the most potently neutralizing mAbs inhibited binding of RRV to Mxra8, a recently discovered alpahvirus receptor. Epitope mapping studies identified the A and B domains of the RRV E2 protein as the major antigenic sites for the human neutralizing antibody response. In experiments in mice, these mAbs were protective against cinical disease and reduced viral burden in multiple tissues, suggesting a potential therapeutic use for humans.

## Author summary

Ross River virus (RRV) was first identified in Australia in 1959, and has since caused multiple outbreaks, some affecting tens of thousands of individuals throughout the Pacific Islands, Australia, and Papua New Guinea. In addition, a mean of 4,600 cases of RRV disease occur in Australia each year. RRV is transmitted to humans via the bite of a mosquito, and disease symptoms include rash, fever, and debilitating polyarthritis. Currently, the adaptive immune response during RRV infection is poorly understood, and no human moncoclonal antibodies (mAbs) against the virus exist. In this study, we generated a panel of human mAbs specific for RRV from two donors who had undergone a natural infection with the virus. We then used these mAbs to elucidate antigenic regions of RRV, and to further study mechanisms by which RRV is neutralized. In addition to potently neutralizing virus *in vitro*, these mAbs significantly reduced mouse death and reducd viral burden in an immunocompromised model. Our study provides new insight into the antibody reponse during a natural infection with RRV, and suggests that therapeutic administration of mAbs may be beneficial in reducing disease burden.

## Introduction

Ross River virus (RRV) is a positive-sense, single-stranded RNA virus in the Alphavirus genus of the *Togaviridae* family. RRV circulates in Australia and Papua New Guinea and is transmitted through the bite of *Aedes* and *Culex* mosquitos. Typical signs and symptoms of infection include rash, fever, and most prominently, debilitating muscle and joint pain that persists for 3 to 6 months [1–7]. RRV is an Australian nationally notifiable disease, and since reporting began in 1993, a mean number of 4,600 cases has been reported in Australia each year [8]. In addition, the economic burden, including diagnosis, treatment and lost wages, has been estimated to be as much as 5 million Australian dollars annually [1,6]. RRV was first isolated from human serum using suckling mice in 1972, but was not connected with symptoms until large epidemics in the South Pacific islands in 1979–1980, in which approximately 70,000 people were infected [1,9,10]. Traditionally, reservoirs of RRV were thought to be maruspials endemic to Australia, such as kangaroos and wallabies [11,12]. However, recent evidence indicates that other mammalian species such as rodents, rabbits, and flying foxes can act as reservoirs for the virus and contribute to its spread [12,13]. This finding suggests that RRV may have the potential to spread to regions outside of Australia and the Pacific Islands and raises concerns about future epidemic transmission. Currently, there are no approved vaccines or specific therapies targeting RRV.

In a previous clinical trial of an experimental inactivated RRV vaccine, immunized individuals produced neutralizing antibodies in serum that conferred protection in mice during subsequent passive transfer studies [14–16]. In addition, several murine mAbs that bind to RRV have been reported, although functional characterization of these mAbs generally has been limited [17–20]. The mature alphavirus glycoprotein is composed of the E1 and E2 envelope proteins in a heterodimer, which is expressed as a trimeric spike on the virus surface after cleavage of the E3 protein by furin-like proteases [21]. Neutralization escape mutants have localized the epitopes of mouse anti-RRV mAbs to the B domain and the flanking region within the E2 glycoprotein [17,18,20]. In comparison, the A and B domains on the E2 glycoprotein and domain II of the E1 glycoprotein of the related alphavirus chikungunya virus (CHIKV) are important targets of neutralizing antibodies [22–24]. However, human monoclonal antibodies (mAbs) specific for RRV have not been reported.

Here, we describe human mAbs isolated from individuals who were naturally infected with RRV. These mAbs neutralized RRV infectivity in cell culture, protected mice when administered therapeutically, and reduced viral burden in multiple tissues. Furthermore, these mAbs bind to multiple domains on the E2 protein, as determined through alanine scanning mutagenesis, and roughly fall into two competition-binding groups, revealing that the A and B domains are the major antigenic targets for the human neutralizing antibody response. These mAbs blocked infection by preventing viral attachment and entry to the cell and also blocked at a later step in the virus lifecyle associated with viral fusion. Notably, nearly all of the neutralizing mAbs blocked attachment of RRV to Mxra8, a recently discovered entry receptor for RRV, CHIKV, and other arthritogenic alphaviruses [25].

## Results

### Isolation of RRV-reactive human mAbs

We isolated a panel of mAbs from two subjects, one with a previous laboratory-confirmed case of RRV that was acquired in Australia in 1987, and the other with a clinical history of childhood infection in Australia in the 1990s. Blood samples were obtained after written informed consent with approval from the Vanderbilt University Medical Center Institutional Review Board from the first donor in 2016 and from the second donor in 2017, and peripheral blood mononuclear cells (PBMCs) were isolated. We transformed B cells with Epstein-Barr virus (EBV) before screening for secretion of RRV-reactive antibodies in cell supernatants through direct virus binding ELISA. We established stable hybridoma cell lines from B cells secreting antibody reactive with the virus and purified 21 mAbs after cloning the cell lines by single-cell flow cytometric sorting. All but one of the mAbs isolated were of IgG1 subclass, and the antibody clonotypes identified by the recombined antibody variable genes were distinct. Two antibodies, designated RRV-130 and RRV-135, had identical variable (V) gene region sequences, but used a different joining (J) gene in the heavy chain (**S1 Table**).

### Assessment of mAb binding and neutralization activity

**Fig 1:**
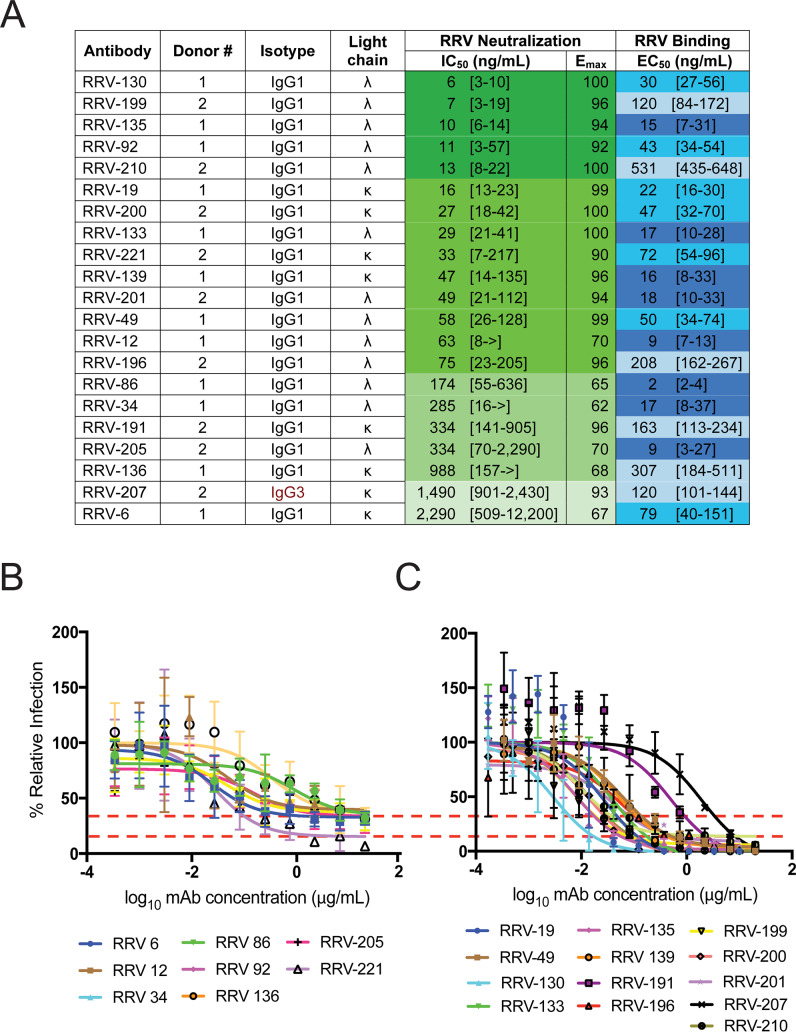
Antibodies generated against RRV bind and neutralize RRV. **(A)** Isotype, subclass, light chain designation (λ or κ), half maximal inhibitory concentration (IC_50_) for neutralization, and half maximal effective concentration (EC_50_) for binding are shown. Binding was measured using ELISA; neutralization was measured using a focus reduction neutralization test (FRNT). Both ELISA and FRNT were performed in triplicate, and 95% credible interval for EC_50_ and IC_50_ values are indicated in brackets. Curves and EC_50_ binding values were obtained using non-linear fit analysis using Prism software version 7 (GraphPad Software). IC_50_ neutralization values were obtained using 5 paramater logistic curves (S1 Methods). Values are color-coded according to binding or neutralization potency, with a stronger binder or neutralization indicated by a darker blue or green color, respectively. > indicates neutralization was not detected, when tested at concentrations up to 10,000 ng/mL. **(B)** Neutralization profiles of mAbs were divided into two groups, based on pattern of activity in a dilutional FRNT assay. Eight antibodies left a resistant fraction of 10 to 40% virus (based on E_max_ value), indicated by the red dotted lines and **(C)** thirteen antibodies completely eliminated virus. Error bars represent SD, and curves are representative of multiple experiments are shown.

The 21 RRV-reactive mAbs were identified by binding to infectious RRV particles in a direct ELISA. Fifteen of these mAbs had half maximal effective concentration (EC_50_) values for binding to RRV of less than 100 ng/mL (**Fig 1A**). When tested for neutralization against the prototype strain RRV T48 in a focus reduction neutralization test (FRNT), five mAbs had half maximal inhibitory concentration (IC_50_) values less than 15 ng/mL, indicating highly potent neutralization in cell culture, whereas nine other mAbs had neutralization IC_50_ values less than 100 ng/mL (**Fig 1A, S2 Table**). Two distinct neutralization profiles were observed for these mAbs: one group of seven mAbs left a residual fraction of non-neutralized virus with a 60 to 90% maximal reduction of infection (**Fig 1B, S1 Fig**), whereas the second group of fourteen antibodies completely eliminated virus infection (**Fig 1C, S1 Fig**). Four antibodies also were tested in neutralization assays using a representative panel of five diverse clinical isolates of RRV and had comparable neutralization potencies compared to the T48 strain (**S2 Fig, S2 Table**).

### Epitope mapping using alanine scanning mutagenesis and competition-binding assays

**Fig 2:**
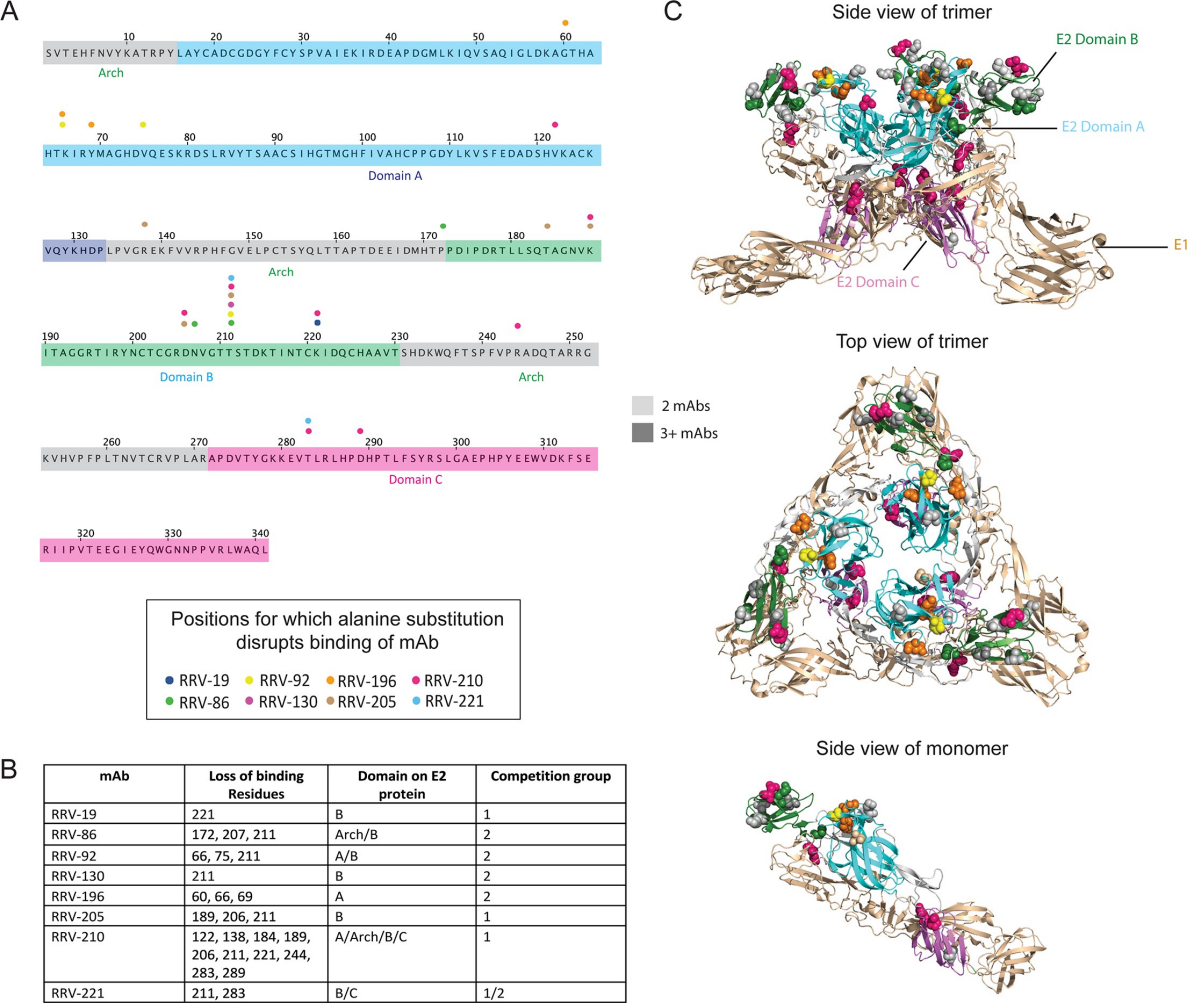
Alanine scanning mutagenesis reveals E2 residues important for mAb binding. **(A)** Amino acid sequence of E2 from the RRV T48 strain, indicating loss-of-binding residues determined through alanine scanning mutagenesis. Each amino acid residue is numbered according to its position within the E2 protein, with the A, B, and C domains along with the arch regions [22,26] color coded (grey, arch; dark blue, domain A; green, domain B; magenta, domain C). A circle above the sequence indicates the position of residues for which alanine substitution disrupts mAb binding, with each circle color corresponding to a different mAb. **(B)** Summary table with residues disrupted by alanine scanning mutagenesis, including the E2 domain in which they are found and the competition group to which the mAb belongs (see **Fig 3**) Two independent experiments were performed and values were averaged for loss-of-binding determination. A cutoff value of 10% was used, with the requirement that two other mAbs have binding of 50% or greater. **(C)** Loss-of-binding residues mapped onto the crystal structure of the CHIKV E1/E2 heterodimer (PDB 3N42), with three heterodimers subunits combined to represent the viral spike trimer. Top and side views of the trimer are shown, with residues important for mAb binding color coded as in (**A**) and shown as space-filling forms. The E2 protein is shown in green and the E1 protein in light brown, and each of the domains is labeled as in (**A**). A side view of a single heterodimer subunit is also shown (bottom).

To identify the antigenic regions recognized by these neutralizing mAbs, we first performed alanine scanning mutagenesis using cell-surface expression of RRV proteins and flow cytometric screening to identify critical binding residues in the E2 glycoprotein. Our library consisted of the first 300 residues of the E2 protein individually mutated to alanine, with alanine residues mutated to serine. We observed a loss of binding for eight mAbs, with residues spread across the A, B, and C domains, as well as arch region of the E2 protein (**Fig 2A, S3 Fig**). RRV-196 had loss-of-binding residues within the A domain alone, whereas RRV-92 and RRV-210 lost binding when residues were changed in the A, B and C domains, as well as arch region, of E2 (**Fig 2A and 2B**). Seven of the eight mAbs targeted regions within the B domain, and of those, three mAbs had loss-of-binding residues within the B domain alone. Within the B domain, residues 189, 206, and 221 showed decreased binding for two mAbs, and mutation of residue 211 resulted in loss-of-binding for six mAbs: RRV-86, RRV-92, RRV-130, RRV-205, RRV-210, and RRV-221 (**Fig 2A and 2B)**. When we mapped these residues onto the surface of the structure of the related CHIKV E1/E2 heterodimer (PDB 3N42) [26], we found that most residues clustered within the surface-exposed region of the viral glycoprotein, with only a few located at the base of the heterodimer subunit in the C domain (**Fig 2C**).

**Fig 3:**
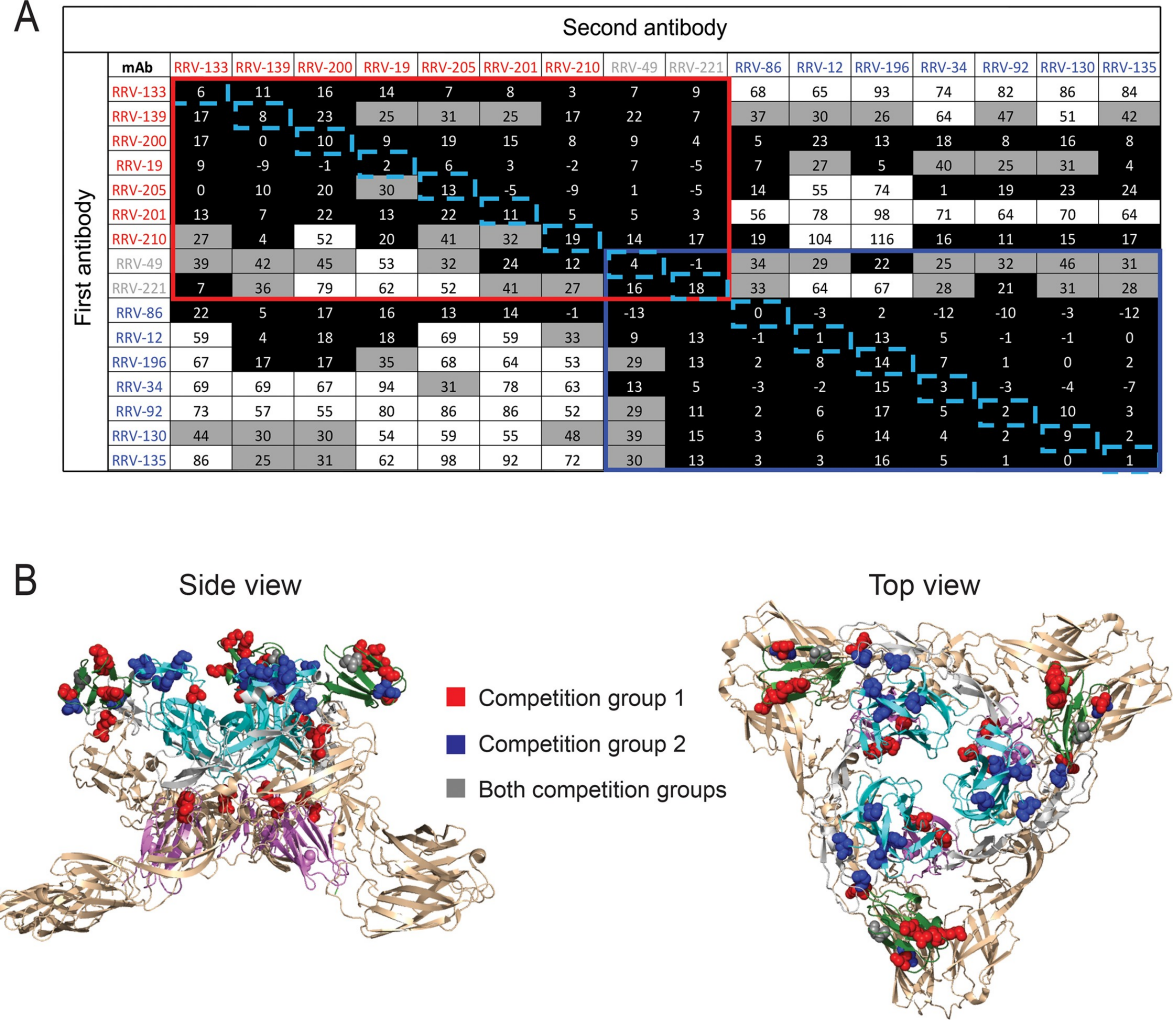
Epitope mapping studies to identify groups of mAbs recognizing similar major antigenic sites. **(A)** An Octet RED96 instrument (Pall FortéBio) was used to perform epitope binning studies using competition binding. RRV-86 was used as a capture antibody and was immobilized onto Fc-specific anti-human IgG biosensors for 2 min. After measuring the baseline signal, the biosensor tips were immersed into wells containing RRV VLPs for two minutes. After another baseline measurement, biosensors then were transferred to wells containing a first mAb at a concentration of 100 μg/mL for 5 min, before immersion in a solution containing a second mAb, also at a concentration of 100 μg/mL for 5 min. The percent competition of the second mAb in the presence of the first mAb was determined by comparing the maximal signal of binding for the second mAb in the presence of the first antibody to the maximal signal of the second mAb in the absence of competition. Competition was defined by reduction of the maximal binding score to <25% of un-competed binding (black boxes). A non-competing mAb was identified when maximal binding was >50% of un-competed binding (white boxes). A 25 to 50% reduction in maximal binding was considered intermediate competition (gray boxes). Some values are negative due to slight dissociation of the first antibody in the presence of the second. The colored boxes denote two overlapping asymmetrical competition groups. The blue dotted boxes highlight antibody self-competition. **(B)** Residues corresponding to mAbs in each competition group as determined through alanine scanning mutagenesis mapped onto the CHIKV E1/E2 trimer of heterodimers (PDB 3N42). Space-filling models for loss-of-binding residues in competition group 1 are shown in red, and loss-of-binding residues for competition group 2 in orange. A top view of the trimer is shown (left) as well as a side view (right).

As another method to distinguish antibody epitopes, we performed a quantitative competition-binding assay using biolayer interferometry (BLI). We used RRV-86 immobilized on Fc-specific anti-human IgG biosensor to capture virus-like particles (VLPs) expressing the full set of structural proteins (C-E3-E2-6K-E1). We then added two antibodies sequentially, and the percent binding of the second antibody in the presence of saturating concentrations of the first antibody was determined. Five out of twenty-one mAbs were excluded from the analysis due to undectable or weak binding to VLPs in this format. Antibodies in the panel binned roughly into two competition-binding groups, with seven mAbs in the first group (red box), seven in the second group (blue boxes), and two in an overlapping group (grey boxes) (**Fig 3A**). The order of antibody addition made a difference in the competition profile, as in some cases the first antibody blocked binding of the second antibody, but the reverse was not true, as in the case of RRV-200 and RRV-34. This effect may be due in part to steric hindrance mediated by Fc regions in the full-length antibody during binding to the VLP. While there was no correlation between whether mAbs were completely neutralizing or not and their competition group, we did observe a correlation between our alanine scanning mutagenesis data and competition data. MAbs in the first competition group (red) showed predominant epitopes within the B domain (green) and C domain (magenta) when overlaid on the CHIKV E1/E2 trimer, whereas those in the second group (orange) mapped primarily to the A domain (dark blue) and arch regions (grey) (**Fig 3B)**. Two mAbs had a competition profile that overlapped between the two groups, and additionally, some residues uncovered through the alanine scanning mutagenesis were targeted by mAbs within both competition groups (grey) (**Fig 3B)**.

### Mechanisms of virus neutralization

**Fig 4:**
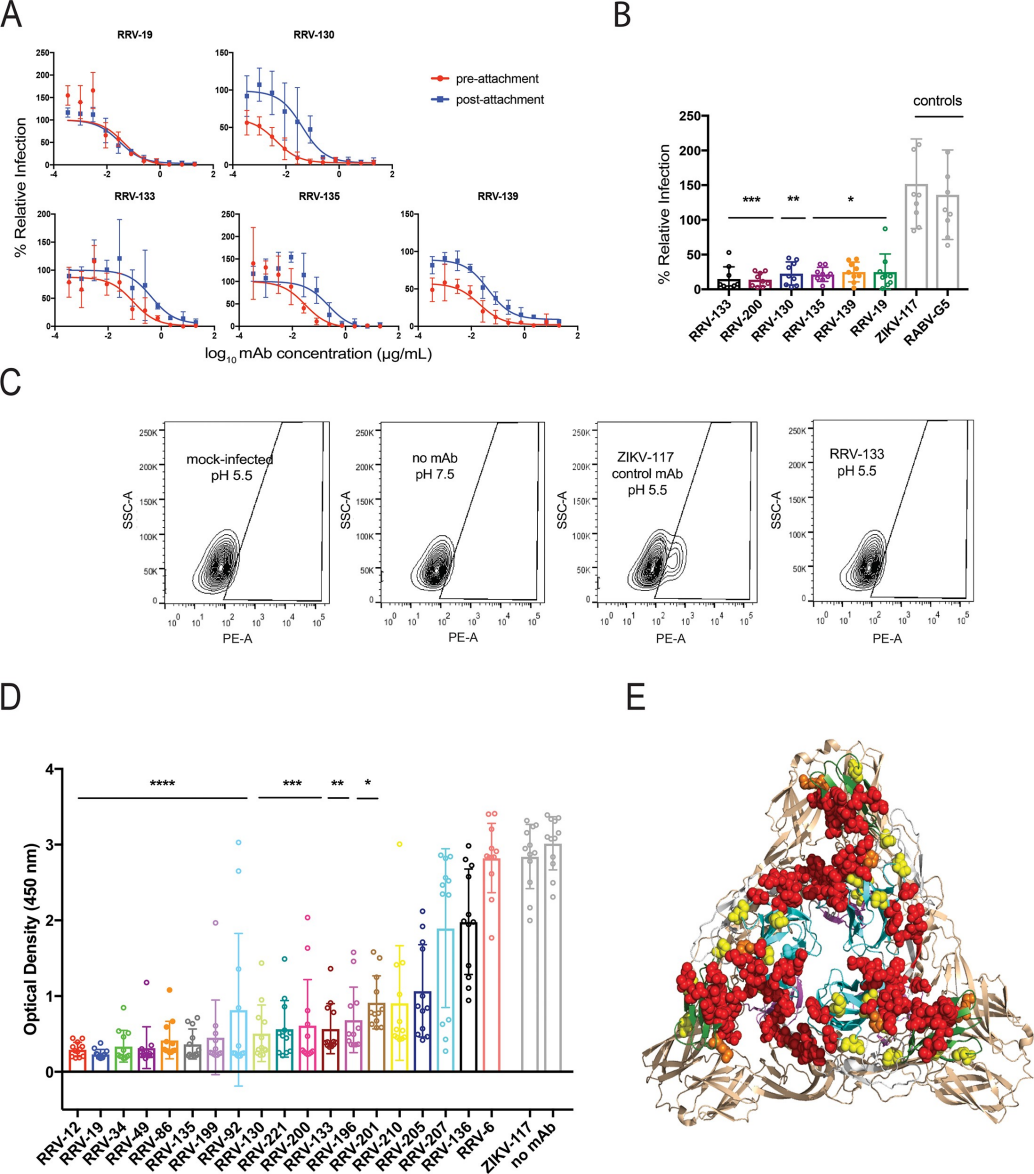
RRV mAbs neutralize through multiple mechanisms. **(A)** Pre-attachment and post-attachment neutralization assays were performed for representative mAbs from each competition group, and a focus-forming assay was used to quantify reduction in infection. In the pre-attachment assay, antibody was incubated with virus at 4°C before addition to Vero cells kept at 4°C. For the post-attachment assay, virus was applied to Vero cell monolayer cultures at 4°C before addition of antibody to cells at 4°C. Two independent eperimnents were performed in triplicates for each antibody, and representative curves are shown. **(B)** A fusion from without (FFWO) assay was used to measure antibody inhibition of virus fusion with the cell membrane under low pH conditions. Virus was adsorbed to Vero cell culture monolayers at 4°C for an hour before addition of antibody dilutions, also at 4°C, after removing excess virus. Cells then were exposed to a pH 5.5 medium or a control medium at neutral pH for two minutes and incubated at 37°C. The acidic pH medium was removed and cells were incubated for an additional 14 h before fixing, permeabilizing, and staining for intracellular virus antigens before flow cytometric analysis. Intracellular virus was quantified by measuring percent PE-positive cells relative to a virus-only control. Three separate experiments were performed in triplicates for each antibody (Kruskal-Wallis one-way ANOVA with Dunn’s post-test, with mean  ± S.D. compared to virus-only control. (*p < 0.05, **p < 0.01, ***p < 0.001)). **(C)** Representative flow cytometry contour plots are shown for the FFWO assay. Mock-infected cells under a low-pH condition and cells with no antibody under a neutral pH condition (to ensure that virus only entered the cell through pH-mediated fusion) are shown as negative controls, and cells with a non-specific mAb (ZIKV-117) under a low pH condition are shown as a positive control. **(D)** Antibody blocking of RRV binding to mouse Mxra8-Fc fusion protein was determined through competition ELISA. Virus was captured on the plate with a human mAb before addition of RRV mAbs followed by Mxra8-mFc (mouse Fc). A loss of signal indicates competition of RRV mAbs with Mxra8-mFc for binding to virus. Three independent experiments were performed in quadruplicate (Kruskal-Wallis one-way ANOVA with Dunn’s post-test, with mean  ± S.D. compared to isotype control (*p < 0.05, **p < 0.01, ***p < 0.001, ****p < 0.0001)). **(E)** Residues that result in loss of Mxra8 binding to cell-surface-displayed chikungunya proteins are mapped onto the CHIKV E1/E2 trimer of heterodimers (PDB 3J2W) in red, and the alanine footprint of mAbs that block binding of mouse Mxra8-Fc protein to RRV are shown in yellow. Overlapping epitopes of RRV mAbs and Mxra8 contact residues are denoted in orange.

To gain insight into the mechanism(s) of neutralization used by these mAbs, we performed pre- and post-attachment neutralization assays with representative mAbs from each of the competition-binding groups. RRV-19, RRV-133, and RRV-139 were chosen from group 1, and RRV-12, RRV-130, and RRV-135 were chosen from group 2. These mAbs have a range of neutralization potencies, and at least one mAb (RRV-12) neutralizes incompletely. In the pre-attachment assay, virus was incubated with antibody at 4°C before addition to Vero cell monolayers, also at 4°C. Virus not attached to the cells and unbound antibody were washed out, and then attached virus was allowed to internalize during a brief incubation period at 37°C. Cell monolayers were stained 18 h later, as in the FRNT. All six mAbs neutralized in the pre-attachment assay, indicating that these mAbs block either cell adherence or entry of virus (**Fig 4A**). In the post-attachment assay, which detects effects both on viral entry and on downstream steps such as fusion from the endosome, virus was adsorbed first to cells at 4°C. Excess virus was washed out before mAb was added, also at 4°C. After a brief incubation period at 37°C to allow virus internalization, cells were overlaid with methylcellulose, incubated, and then fixed and stained 18 h later. All mAbs also blocked at post-attachment steps, although post-attachment neutralization was slightly less potent than pre-attachment for RRV-130, RRV-133, RRV-135, and RRV-139 (**Fig 4A**). As in the FRNT described previously, RRV-12, the only incompletely neutralizing mAb chosen for further characterization, did not completely eliminate viral foci in either of these mechanistic neutralization assays, leaving a residual fraction of ~25% compared to untreated virus.

To determine if some of the inhibitory activity in post-attachment neutralization was due to antibody-mediated prevention of RRV fusion to cell membranes after entering the endosome, we performed a fusion from without (FFWO) assay. This assay, which measures fusion of virus with the plasma membrane under low pH conditions, has been used as a surrogate assay for determining antibody inhibition of alphavirus fusion in endosomes [22,24,27]. Virus was absorbed first to cells at 4°C before mAbs were added. Subsequently, after removing unbound virus and antibody, cells were pulsed at 37°C in a low-pH medium to promote plasma membrane-mediated viral fusion. Virus that entered the cells was stained with fluorescent antibodies 14 h later and detected by flow cytometry. At a concentration of 10 μg/mL, RRV-133, RRV-130, RRV-135, RRV-139, and RRV-19 significantly reduced virus entry to cells under low-pH conditions (**Fig 4B and 4C**). RRV-12 did not inhibit fusion, with virus levels comparable to those of the negative control antibodies. Antibodies in both competition-binding groups inhibited fusion, so inhibition of fusion by mAbs does not seem to correlate with mapping to the A or B domain.

Recently, the cell surface protein Mxra8 was identified as a receptor for CHIKV and several other arthritogenic alphaviruses, including RRV, Mayaro, and o’nyong’nyong viruses [25]. To determine whether the RRV mAbs can block attachment of virus to this receptor, we performed a competition ELISA in which RRV particles were captured onto the plate, and then mAbs were bound to the virus before addition of a purified recombinant mouse Mxra8-Fc fusion protein. MAbs from each of the competition-binding groups blocked binding of Mxra8-Fc binding to RRV (**Fig 4D**). All mAbs that poorly blocked binding to Mxra8 had IC_50_ values of greater than 100 ng/mL, suggesting that neutralization potency might be related to effectiveness of Mxra8 blocking. However, we did not observe a difference in potency of Mxra8 blocking for those mAbs that did or did not leave a resistant fraction of virus in the neutralization assay. Based on our alanine mutagenesis data, the mAbs that blocked binding to Mxra8 contacted residues 60, 66, 69 and 75 in the A domain and residues 172, 207, 211, and 221 in the arch and B domain. We overlaid these residues (yellow) on the CHIKV E1/E2 heterodimer along with the known Mxra8 contact residues on E2: 5–6, 18, 26–29, 62–64, 71–72, 74–76, 119–121, 123, 144, 150, 157–160 178–182, 189, 191–193, 212–214, 221–223, 263–265, 267 (red) [25,28,29], showing that the footprint for these Mxra8-blocking mAbs indeed was in close proximity to the binding footprint for the Mxra8 receptor (**Fig 4E**).

### Therapeutic activity of mAbs *in vivo*

**Fig 5:**
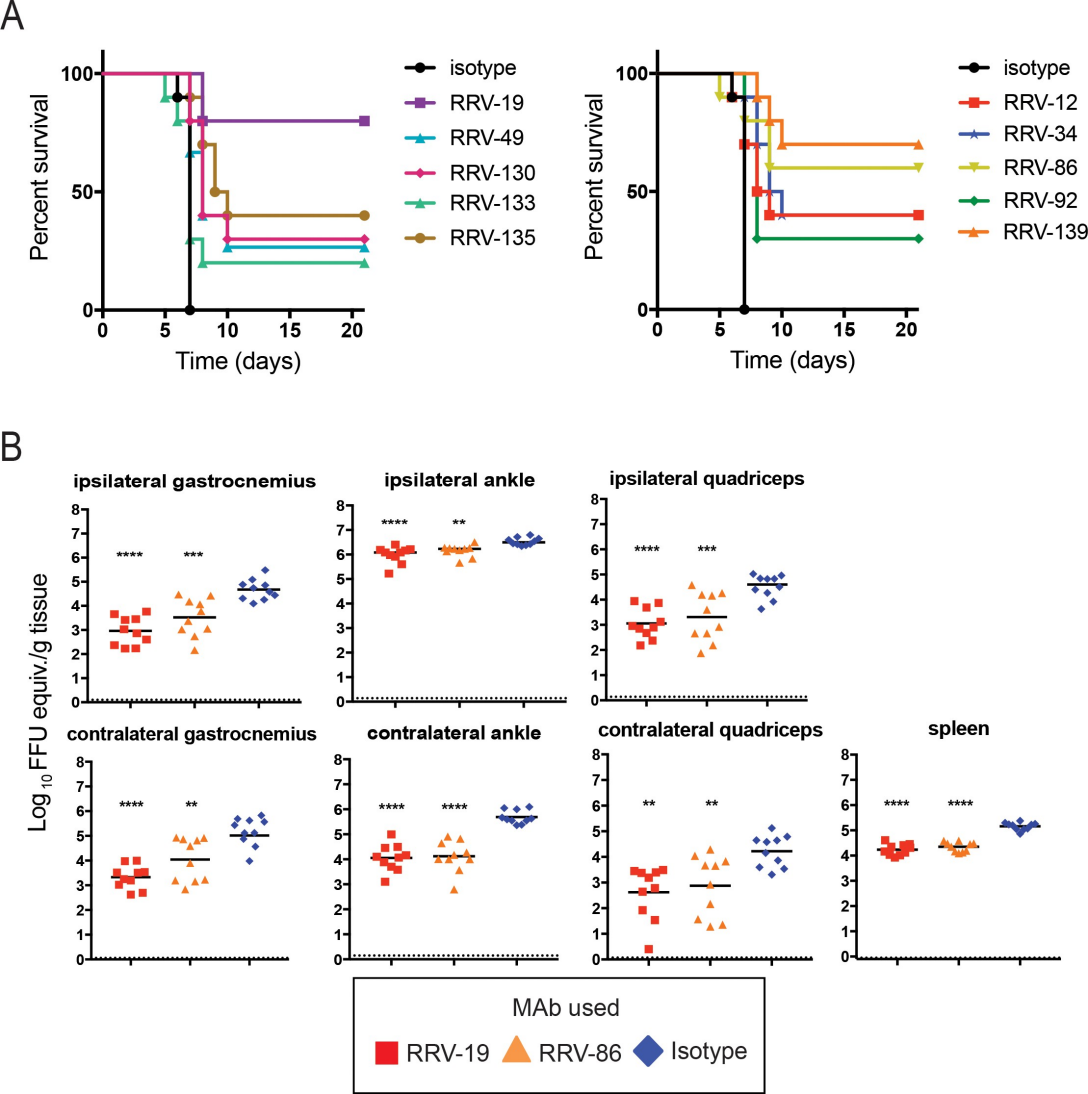
RRV mAbs increase mice survival rates when administered therapeutically. **(A)** WT C57BL/6 mice were given 0.2 mg of anti-Ifnar1 mAb, and subsequently inoculated with 10^3^ FFU of RRV in the footpad. At day one post-infection, 100 μg of RRV mAb was administered. Antibodies are grouped according to neutralization profiles, with those exhibiting incomplete neutralization *in vitro* on the left and those exhibiting complete neutralization *in vitro* on the right. Two independent experiments were performed, with a total of n = 10 for each antibody group. The isotype control in the two graphs are the same. Statistical analysis was performed using a log rank test with Bonferroni correction: RRV-12, p = 0.0057; RRV-19, p < 0.0001; RRV-34, p < 0.0001; RRV-49, p = 0.0038; RRV-86, p = 0.0021; RRV-92, p = 0.0013; RRV-130, p = 0.0004; RRV-133, p = 0.3771; RRV-135, p < 0.0001; RRV-139, p < 0.0001. **(B)** 100 μg of RRV-19, RRV-86, or an isotype control were administered 24 h post-infection to WT immunocompetent C57BL/6 mice, and the ipsilateral and contralateral gastrocnemius, quadriceps, ankle, or spleen tissues were collected 3 dpi. Viral RNA was quantified through qRT-PCR. Two independent experiments were performed, with a total of n = 10 mice for each antibody group (one-way ANOVA with a Dunnett’s post-test comparing each group to the isotype control; **p < 0.01, ***p < 0.001, ****p < 0.0001).

We selected ten antibodies with neutralization IC_50_ values below 100 ng/mL to test for protection in a highly susceptible, immunocompromised mouse model of RRV infection and disease. Four-week old male wild-type (WT) C57BL/6 mice were treated with 0.2 mg of MAR1-5A3 (a blocking anti-Ifnar1 mAb) [30] prior to inoculation with 10^3^ FFU of RRV, and 100 μg of RRV mAb was administered via the intraperitoneal route 24 h after virus inoculation. When given a control mAb for treatment, all mice died after 7 days. In contrast, when given an RRV mAb, mortality was delayed over the course of three weeks (**Fig 5A**). The most effective mAb, RRV-19, protected 80% of mice, followed by RRV-139 at 70% protection, and RRV-86, at 60% protection. We did not detect a difference in protection between those mAbs that left a resistant fraction of virus (left) and those that neutralized completely (right), although the two most protective mAbs neutralized completely (**Fig 5A**).

Since RRV infection in humans is rarely fatal, we tested RRV-19 and RRV-86, representing mAbs from different competition-binding groups, in an immunocompetent mouse model of RRV-induced myositis where infection results in high viral burden in muscles and joint-associated tissues [31]. Four-week old male WT C57BL/6 mice were treated with mAbs 24 hpi with 10^3^ FFU of RRV. The gastrocnemius (calf muscle), ankle, spleen, and quadriceps were harvested 3 dpi and viral RNA burden was measured by qRT-PCR. Although both mAbs significantly reduced viral RNA in all tissues, RRV-19 was more effective in the ipsilateral and contralateral gastrocnemius, as well as the ipsilateral ankle and quadriceps (**Fig 5B**). RRV-19 and RRV-86 were equally effective in the contralateral ankle and quadriceps as well as the spleen. While there was less than a 10-fold decrease in viral RNA burden in the ipsilateral ankle, this difference was significant (**Fig 5B**).

**Fig 6:**
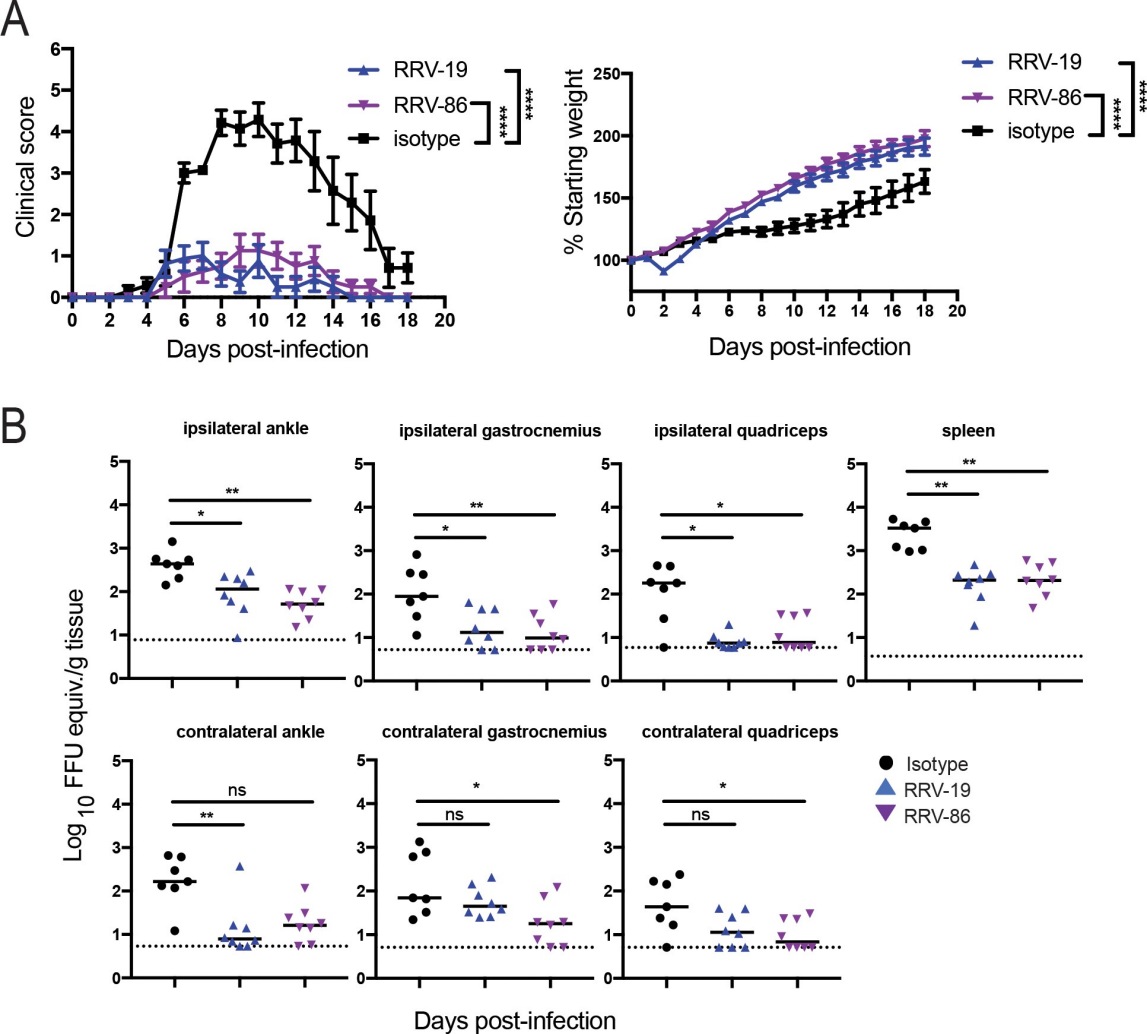
RRV mAbs improve clinical disease and reduce viral RNA burden when given therapeutically in a WT mouse model. **(A)** Three-week-old WT C57BL/6 mice were inoculated with 10^3^ FFU of RRV strain T48 before administration of 100 μg antibody by intraperitoneal injection at 24 hpi. Mice were then weighed each day over the course of 18 days and assigned a clinical score based on grip strength, gait, and righting reflex. Blind scoring of mice was performed using the following scoring system: 0, no disease; 1, mild defect in ipsilateral hind paw gripping; 2, mild defect in bilateral hind paw gripping; 3, bilateral loss in hind paw gripping; 4, bilateral loss in hind paw gripping with moderate hind limb weakness, observable mild altered gait, and difficulty or failure to right self; 5, bilateral loss in hind paw gripping with severe hind limb weakness, moderate altered gait, and loss of righting reflex; 6, bilateral loss in hind paw gripping with severe hind limb weakness, severely altered gait with possible dragging hind paw, and loss of righting reflex; 7, moribund. Two independent experiments were performed, for a total of n = 8 mice in each antibody group. Statistical analysis was performed using a one-way ANOVA of area under the curve test (****p < 0.0001). **(B)** Eighteen days post-infection, the spleen, ipsilateral and contralateral gastrocnemius, quadriceps, and ankle tissues were collected following extensive perfusion with PBS. Viral RNA was quantified through qRT-PCR and statistical analysis was performed using a Kruskal-Wallis multiple comparisons test (*p < 0.05, **p < 0.01; ns = not significant).

In addition to measuring viral RNA burden in select tissues, we also measured severity of RRV disease in the immunocompetent model using a clinical scoring system. In this study, three-week-old WT C57BL/6 mice were inoculated with 10^3^ FFU of RRV strain T48 before intraperitoneal administration of 100 μg of RRV-19, RRV-86, or an isotype control mAb at 24 hpi. Mice were then weighed and a clinical score was assigned based on grip strength, gait, and righting reflex, as previously described [32]. All mice receiving the anti-RRV mAbs were protected from weight loss and clinical disease, as compared to mice given the isotype control (**Fig 6A**). In addition, viral RNA was quantified after harvest of the spleen, ipsilateral and contralateral gastrocnemius, quadriceps, and ankle tissues 18 dpi. A significant reduction in viral RNA was observed for administration of all mAbs except for RRV-19 in the contralateral calf and quad (**Fig 6B**).

## Discussion

These studies provide insight into the antibody response for RRV, using human mAbs isolated from naturally infected donors. The isolated mAbs recognize epitopes within the E2 glycoprotein, potently neutralize the RRV laboratory prototype strain in addition to five clinical isolates, and treat infection *in vivo* in a mouse model by reducing viral dissemination. We showed that a subset of mAbs neutralize at both pre-attachment and post-attachment stages, indicating that there are multiple mechanisms of neutralization for these mAbs. Additionally, nearly all neutralizing antibodies blocked binding of the receptor Mxra8 to RRV *in vitro*, and many also inhibited viral fusion with cell membranes. The majority of these mAbs had neutralization IC_50_ values less than 100 ng/mL, and several exhibited highly potent (< 15 ng/mL) neutralizing activity, which is similar in range to the best-in-class human neutralizing antibodies for the related CHIKV [22]. For nearly half of these RRV mAbs, a 10 to 40% residual fraction of virus was not inhibited in neutralization assays. The molecular basis for this residual fraction of infectious virus remains unclear but could reflect particle heterogeneity due to maturation or incomplete release of the E3 precursor protein [33,34].

Through alanine scanning mutagenesis, we discovered multiple epitopes for these mAbs scattered across the A and B domains and the arch region, of the E2 protein. Because our alanine library only consisted of residues within the E2 protein, we did not test whether some mAbs contact the E1 protein. Two main antigenic regions emerged between residues 60–75 in the A domain of E2 and 206–221 of the B domain. A major antigenic region has been suggested previously for RRV using three murine mAbs [17,18]. These antibodies were localized to the B domain and the adjacent arch region of the E2 protein, between residues 200 and 262, the position of two N-linked glycosylation sites [35]. The epitope for one of these mouse mAbs is at position 216 of the B domain, which is within five residues of 221 and 211, two residues that were targeted by two or more of our human mAbs [18]. Additionally, residues 206 and 207 within the B domain appeared important for binding in our alanine scanning mutagenesis study. Residue 244, which is close to a mouse mAb epitope at position 246 within the arch region connecting domains B and C, also emerged as a recognition site for one of our mAbs. Based on competition-binding studies, we assigned neutralizing antibodies into two major groups with similar profiles. Some asymmetry of competition was present in these groups, and the profiles of two mAbs overlapped between the two groups. This asymmetry may be due to the use of VLPs in competition-binding assays, which better mimic the conformation of the virus than recombinant proteins. While these two competition groups did not correlate perfectly with our alanine mutagenesis data, the majority of the mAbs in group 1 corresponded to residues within the B or C domains, whereas those in group 2 mapped mostly to domain A or the arch region between domains A and B. Although the C domain is not surface-exposed, it is probable that residues within this region may stabilize the B domain, since the B domain undergoes a conformational change to uncover the fusion loop on the E1 protein after virus entry into the endosome [36–38]. Thus, mutating residues in this “hinge” region of domain C could affect mAb binding to domain B allosterically. Previously, neutralizing CHIKV antibodies have been shown to target analogous sites within the A, B, and arch regions of the E2 protein [20,22,27]. Our results indicate that both the A and B domains also are important antigenic sites for the human neutralizing antibody response directed against RRV. The knowledge of immunodominant sites on the virus recognized by potent neutralizing antibodies to RRV has implications for immunogen design, as there is currently no licensed vaccine available.

RRV is thought to have several possible receptors, including α1β1 integrin, which is nearly universally expressed on adherent cells, and Mxra8, a recently discovered receptor for multiple arthritogenic alphaviruses [25,39]. A candidate receptor binding site could span across surface-exposed regions of both the A and B domains, as is hypothesized for Mxra8 [25]. The fact that mAbs from both competition groups with epitopes in the A and B domains of the E2 protein inhibited binding to Mxra8 protein, supports this hypothesis. Several regions of the E2 protein that resulted in loss of Mxra8 binding to cell-surface-displayed CHIKV proteins are residues 62–64, 71–72, and 74–76 [25,28,29], which are close to loss-of-binding residues 66, 69, and 75, determined for Mxra8-blocking mAbs RRV-92 and RRV-196. Additionally, residues 207, 211, and 221 in the B domain of the E2 protein, which were revealed in our alanine scanning mutagenesis as important for binding of RRV-19, RRV-86, RRV-92, RRV-130, and RRV-221, are in close proximity to residues 212–214 and 221–223 that are additional contact sites for Mxra8 [25,28,29].

In addition to attachment/entry inhibition and blockage of binding to Mxra8 receptor, we also observed post-attachment inhibition activity for a subset of mAbs tested, although in most cases, neutralization appeared to be slightly more potent in the pre-attachment assays. During alphavirus infection of a cell under normal conditions, a decrease in pH within the endosome exposes the fusion loop on the E1 protein, which is usually shielded by the B domain of E2 [36–38]. While specific alanine scanning mapping data was not obtained for all of our fusion blocking mAbs, it is possible that these antibodies, which are from multiple competition-binding groups, stabilize the B domain to prevent uncovering of the fusion loop.

Therapeutic studies in mice with an acquired deficiency of type I IFN signaling revealed that all mAbs increased survival of the mice to some degree, and administration of each of three mAbs resulted in a greater than 50% survival rate. Importantly, two mAbs chosen for testing in immunocompetent WT mice significantly improved clinical disease symptoms, as demonstrated by dramatically improved hind limb strength and hind paw gripping ability, as well as mitigation of weight loss. Analysis of total amount of viral RNA present in several tissues showed that both mAbs tested reduced viral RNA burden significantly in nearly all tissues examined. A combination mAb therapy has proven efficacious for CHIKV [24], so it is possible that pairing antibodies such as RRV-19 and RRV-86, which are from different competition-binding groups, could reduce viral burden to a greater degree and prevent emergence of resistance. Additionally, since these mAbs have therapeutic benefit, they could have a protective effect when administered prophylactically, as was seen with CHIKV mAb studies [22,24,40]. While the market for an RRV prophylactic or therapeutic antibody remains small, recent advances in delivery of mRNA-encoded antibodies have potential to greatly decrease manufacturing costs currently associated with a protein therapy [41]. Such an mRNA therapy has already shown promise in mice and macaques for CHIKV [42,43].

A future challenge that must be addressed before clinical use of anti-RRV mAbs is time of administration, since RRV disease currently is diagnosed using paired serology, typically 2 to 3 weeks after onset of symptoms [44]. Our mouse models have demonstrated efficacy of mAbs during the period of peak viremia; however, administration of mAbs to patients within this time window would necessitate more rapid diagnostic procedures, such as virus identification through qRT-PCR. Reduction of viral load in the early phases of disease might not only reduce disease severity, but also might decrease likelihood of human-mosquito transmission. Outbreaks of polyarthritis caused by RRV have been observed recently among Australian military personnel, in which human-mosquito-human transmission was thought to have occurred without an intermediate host [45]. Such transmission highlights another potential clinical use for an RRV mAb, in which travelers or deployed military personnel returning from an epidemic area could be given mAb therapeutically to reduce likelihood of virus transmission to mosquitoes in their local area or country. Therefore, despite challenges associated with mAb therapy, further study is warranted to understand how these mAbs might best be used for treatment or prevention of RRV disease.

## Methods and materials

### Source of human B cells

The first research subject was a 50-year old woman living in the U.S. who had a history of laboratory-confirmed infection in Australia in 1987. The second subject was a 32-year old male who was exposed to the virus during childhood in Australia in a clinically diagnosed but non-laboratory confirmed case of infection. Peripheral blood was obtained from the first donor in 2015 (28 years after infection) and from the second donor in 2017 (approximately 20 years after infection) with written informed consent following approval of the study by the Vanderbilt University Medical Center Institutional Review Board. Peripheral blood mononuclear cells (PBMCs) were isolated from both donors using density gradient centrifugation on Ficoll and were cryopreserved in liquid nitrogen until used in the experiments.

### Generation of human hybridomas

Approximately 10 million cryopreserved PBMCs were thawed and transformed with Epstein-Barr virus obtained from the supernatant of B95.8 cells in a suspension also containing a Chk2 inhibitor, cyclosporin A, and CpG, and the mixture was plated in a 384-well cell culture plate. After 7 days, transformed cells in each well were transferred to a well in 96-well plates containing a feeder layer of irradiated cells that were PBMCs obtained from discarded leukofiltration devices (Nashville Red Cross). After an additional 5 days, the supernatants of expanded cells were screened for the presence of RRV-reactive antibodies using an enzyme-linked immunosorbent assay (ELISA), described below. Transformed B cells from wells containing supernatant with antibodies reactive to RRV were fused to the HMMA2.5 non-secreting myeloma cell line using an established electrofusion technique [46]. After fusion, the resulting mixture of hybridoma cells was resuspended in medium containing hypoxanthine, aminopterin, thymidine and oubain to select for hybrids of B cells and myeloma cells.

### RRV ELISA screen

RRV strain T48 was propagated in monolayer cultures of Vero cells. The cell line was authenticated and tested monthly during culture for the presence of mycoplasma and found to be negative in all cases. Infected cell supernatants containing virus with a titer of approximately 5 x 10^6^ FFU/mL were harvested when cytopathic effect was maximal, filtered through a 0.45 μm filter, then frozen and stored at –80°C until use. 384-well ELISA plates were coated with 25 μL of RRV strain T48 diluted 1:100 in PBS, and incubated for 1 h at 37°C. Plates were washed 5 times with PBS containing Tween (PBST) using an EL406 combination washer dispenser instrument (BioTek) and blocked for 1 h at room temperature with 5% milk powder and 2% goat serum, diluted in PBS. After washing 2 times with PBST, 25 μL of supernatants from hybridoma cultures or EBV-transformed cell lines were added to plates, which then were incubated for 1 h at room temperature. Plates were washed 4 times, and 25 μL of goat anti-human alkaline phosphatase-conjugated secondary antibodies (Meridian Life Science) diluted 1:5,000 in PBS was added to plates. After a 45-min incubation period, plates were washed 5 times and 25 μL of alkaline phosphatase substrate tablets (Sigma) diluted in Tris buffer with 1M MgCl_2_ was added. Optical density was read at 405 nm after 1 h using a Biotek plate reader.

### Biolayer interferometry (BLI) competition-binding studies

An Octet RED96 BLI instrument (Pall FortéBio) was used to perform epitope binning studies using competition binding. One of the human antibodies obtained in early experiments (RRV-86) was used as a capture antibody and was immobilized onto Fc-specific anti-human IgG biosensors for 2 min. After measuring the baseline signal, the biosensor tips were immersed into wells containing RRV VLPs for 2 min. After another baseline measurement, biosensors then were transferred to wells containing a first mAb at a concentration of 100 μg/mL for 5 min, before immersion in a solution containing a second mAb, also at a concentration of 100 μg/mL for 5 min. The percent competition of the second mAb in the presence of the first mAb was determined by comparing the maximal signal of binding for the second mAb in the presence of the first antibody to the maximal signal of that mAb alone when separately tested uncompeted. Competition was defined by reduction of the maximal binding score to <20% of un-competed binding. A non-competing mAb was identified when maximal binding was >50% of un-competed binding. A 25 to 50% reduction in maximal binding was considered intermediate competition.

### Generation of virus-like particles (VLPs)

RRV structural genes, capsid-E3-E2-6K-E1, encoding 3,783 bp with the addition of a Kozak sequence, were cloned into the pcDNA3.1(+) mammalian cell expression plasmid (GenScript). 293T cells (American Type Culture Collection [ATCC] Cat. No. CRL-11268) were transfected with 4 μg per 5 ×10^5^ cells of the plasmid using the Lipofectamine 2000 method according to the protocol of the manufacturer (Thermo Fisher Scientific). Transfection was allowed to proceed for 48–72 h before supernatant was harvested and filtered through a 0.45 μm filter. VLPs were concentrated by ultracentrifugation at 110,000 g in a SW28 rotor for 2 h at 4°C through a 20% sucrose cushion using a Sorvall Discovery 90SE ultracentrifuge. The resulting pellet was resuspended in 250 μL of TNE buffer (0.01 M Tris-HCl, pH 7.2, 0.1 M NaCl, 0.001 M EDTA) and stored at 4°C.

### Hybridoma cell line clone production

Two weeks after fusion, hybridoma cell lines were cloned by single-cell sorting using fluorescence-activated cell sorting on a BD FACSAria III sorting cytometer with aerosol containment, in the Vanderbilt University Medical Center Flow Cytometry Core. Approximately 2 weeks later, an ELISA screen was performed, and wells containing cloned cells secreting antibodies reactive to RRV were selected for expansion.

### Purification of mAb IgG protein

Clonal cells secreting mAbs were grown in 75 cm^2^ flasks to 70% confluency in hybridoma growth medium (ClonaCell-HY medium E from STEMCELL Technologies, 03805). The cells were expanded equally to four 225 cm^2^ flasks for antibody expression in serum-free medium (GIBCO Hybridoma-SFM, Invitrogen, 12045084). The supernatant was harvested after 3 weeks and purified by affinity chromatography using protein G columns (GE Life Sciences, Protein G HP Columns). Purified IgG from hybridoma cell expression was used for all assays.

### Focus reduction neutralization test

Vero cells (American Type Culture Collection [ATCC] Cat. No. CCL-81) were seeded in 96-well plates at 30,000 cells/well the day before use. Antibodies were diluted in 96-well U-bottom plates, with a 1:3 dilution series across the plate and a virus-only control in the left column. A solution containing infectious RRV was diluted to a concentration of 100 focus-forming units (FFU)/mL and mixed 1:1 by volume in a 96-well plate with antibody suspensions. The virus/antibody mixture was incubated for 1 h at 37°C before transfer to Vero cell monolayer cultures. Infection was allowed to proceed for 1.5 h and then 1% methylcellulose overlay prepared in DMEM with 2% FBS was added to cells. After 18 h, 1% paraformaldehyde (PFA) prepared in PBS was used to fix cells for at least one hour. Plates were washed 3 times with PBS before addition of a 1:6,000 dilution of anti-RRV mouse ascites fluid (ATCC Cat. No. VR-1246AF) prepared in cell permeabilizing buffer (PBS with 0.1% saponin and 0.1% bovine serum albumin). After incubation for at least 2 h at room temperature, plates were washed 3 times in permeabilizing buffer, and anti-mouse HRP-conjugated secondary (Kirkegaard & Perry Laboratories [KPL]) was added at a 1:2,000 dilution in permeabilizing buffer. Plates were incubated for 1 h at room temperature, and washed 3 times before addition of TrueBlue Peroxidase substrate (KPL) for 20 min. Plates were rinsed with dH_2_O, and then plates were imaged with an ImmunoSpot plate reader (Cellular Technology Limited [CTL]). Foci were counted with BioSpot 5.1 software (CTL), and the percent relative infection was calculated based on the virus-only control. Triplicate tests were performed for each antibody, and the results were averaged.

### Fusion from without (FFWO) assay

Vero cells were seeded at 30,000 cells/well in 96-well plates the day before use in the assay. Before the start of assay, cells were washed once with binding medium (RPMI 1640, 0.2% BSA, 10 mM HEPES pH 7.4, and 20 mM NH_4_Cl) at 4°C, and incubated for 15 min at 4°C. The T48 strain of RRV was concentrated to 10^8^ FFU/mL using 100 kDa centrifugal filters (Amicon) Centricon. Virus was prepared in binding medium and added to cells at an multiplicity of infection (MOI) of 15 for 1 h at 4°C. Any remaining free virus was removed with two washes in binding medium. Antibodies were prepared in DMEM containing 2% FBS at 10 μg/mL concentrations and added to cells for 1 h at 4°C. Antibody was removed and fusion with the plasma membrane was initiated by the addition of fusion media (RPMI 1640, 0.2% BSA, 10 mM HEPES, and 30 mM succinic acid at pH 5.5) for 2 min at 37°C. Binding medium (RPMI 1640, 0.2% BSA, 10 mM HEPES at pH 7.4) was used in place of low pH fusion medium in controls wells to ensure that virus entry into cells only occurred due to pH-dependent plasma membrane fusion. After a 2-min incubation, medium was removed and cells were incubated in DMEM supplemented with 5% FBS, 10 mM HEPES, and 20 mM NH_4_Cl (pH 7.4). Fourteen hours later, cells were detached with trypsin, fixed with 1% PFA in PBS for 1 hour, and permeabilized with 0.1% saponin detergent solution. For staining prior to flow cytometry analysis, cells were incubated sequentially with RRV mouse ascites fluid (1:6000 dilution ATCC Cat. No. VR-1246AF) for 1 h and PE conjugated goat anti-mouse IgG secondary antibody for 1 h (ThermoFisher). Cells were analyzed on BD Fortessa flow cytometer with FlowJo software.

### Alanine scanning mutagenesis for epitope mapping

The same construct used to generate virus-like particles (see above) was used to construct an alanine mutation library for mapping of antibody epitopes. The first 300 residues in the RRV E2 protein were mutated to alanine, and alanine codons were mutated to serine and synthesized as cDNA (Twist Bioscience). Each mutant was sequence verified and expressed on the surface of 293F cells for screening using an iQue high-throughput flow cytometer (Intellicyt). Loss of binding for each mAb was determined by measuring reduction in fluorescent signal as compared to signal in cells expressing WT protein. To differentiate loss of binding from absence of protein expression, at least two control antibodies with binding at greater than 50% were required for each residue. A cutoff value of less than 10% binding when normalized to WT was set to determine a loss of binding at each residue. An untransfected cell control for each antibody also was used to ensure specificity of binding.

### ELISA-based Mxra8–Fc competition binding assay

RRV-86 (2 μg/mL) was diluted in PBS and immobilized onto a 384-well ELISA plate before incubation for 1 h at 37°C. The plate was washed four times with PBS containing Tween (PBST) using an EL406 combination washer dispenser instrument (BioTek) and blocked for 1 h at room temperature with 5% milk powder and 2% goat serum, diluted in PBS. RRV T48 strain was diluted to 4 x 10^7^ FFU/mL in PBS and 25 μL per well was added for 1 h at room temperature. After washing five times with PBST, RRV mAbs were diluted to 20 μg/mL in PBS and 25 μL of mAb was added to each well, except for control wells where just PBS was added. Blocking mAbs were incubated for 30 min at room temperature and were left in when 25 μL of Mxra8-Fc (mouse Fc region) [25] fusion protein at a concentration of 10 μg/mL was added to each well. After incubation at room temperature for an hour, the plate was washed four times with PBST and 25 μL per well of a goat anti-mouse HRP-conjugated anti-mouse Fc secondary antibody (SeraCare) was added at a 1:2,000 dilution. After five washes with PBST, plates were developed with TMB Substrate (ThermoFisher Scientific) and the reaction was stopped with H_2_SO_4_. Absorbance was read at 450 nm with a Biotek plate reader. A similarly prepared human mAb specific for Zika virus (ZIKV-117 [47]) was included as a negative control antibody.

### Mouse studies

All animal experiments and procedures were carried out in accordance with the recommendations in the Guide for the Care and Use of Laboratory Animals of the National Institutes of Health. The protocols were approved by the Institutional Animal Care and Use Committee at the Washington University School of Medicine (Assurance number A3381-01). Injections were performed under anesthesia that was induced and maintained with ketamine hydrochloride and xylazine, and all efforts were made to minimize animal suffering.

#### Survival studies

Four-week-old male WT C57BL/6 mice were treated with 0.2 mg of MAR1-5A3 (anti-Ifnar1 antibody) prior to inoculation with 10^3^ FFU of WT RRV T48 strain in the footpad. The following day, 100 μg of RRV antibody or an isotype control antibody to an unrelated viral target was administered to mice by intraperitoneal injection. Mice were observed over the course of 21 days for survival and moribund mice were euthanized.

#### Acute virological studies

Four-week-old WT C57BL/6 mice were inoculated with 10^3^ FFU of RRV strain T48 and then 24 hpi were given 100 μg of antibody by intraperitoneal injection. Three days post-infection, the ipsilateral and contralateral gastrocnemius, quadriceps, and ankle tissues were collected as well as the spleen following extensive perfusion with PBS. RNA was isolated from tissues using the RNeasy mini kit (Qiagen). Viral RNA was quantified by qRT-PCR using the TaqMan RNA to C_T_ one-step kit (Applied Biosystems) with nsp3 specific primers (Forward: 5′- GTG TTC TCC GGA GGT AAA GAT AG -3′, Reverse: 5′- TCG CGG CAA TAG ATG ACT AC -3′)and probe (5′- /56-FAM/ACC TGT TTA/ZEN/CCG CAA TGG ACA CCA/ 3IABkFQ/ -3′) and compared to RNA isolated from viral stocks as a standard curve to determine FFU equivalents.

#### Clinical scoring studies

Three-week-old WT C57BL/6 mice were inoculated with 10^3^ FFU of RRV strain T48 and then 24 hpi were given 100 μg of antibody by intraperitoneal injection. Mice were weighed and assigned a clinical score based on grip strength, gait, and righting reflex, as previously described[32]. Mice were scored blinded and as follows: 0, no disease; 1, mild defect in ipsilateral hind paw gripping; 2, mild defect in bilateral hind paw gripping; 3, bilateral loss in hind paw gripping; 4, bilateral loss in hind paw gripping with moderate hind limb weakness, observable mild altered gait, and difficulty or failure to right self; 5, bilateral loss in hind paw gripping with severe hind limb weakness, moderate altered gait, and loss of righting reflex; 6, bilateral loss in hind paw gripping with severe hind limb weakness, severely altered gait with possible dragging hind paw, and loss of righting reflex; 7, moribund. No mice received a score of 7 throughout the course of the experiment. Eighteen days post-infection, the spleen, ipsilateral and contralateral gastrocnemius, quadriceps, and ankle tissues were collected following extensive perfusion with PBS. Viral RNA was quantified as described above.

### Ethics statement

Peripheral blood was obtained from the first donor in 2015 (28 years after infection) and from the second donor in 2017 (approximately 20 years after infection) with written informed consent following approval of the study by the Vanderbilt University Medical Center Institutional Review Board. All animal experiments and procedures were carried out in accordance with the recommendations in the Guide for the Care and Use of Laboratory Animals of the National Institutes of Health. The protocols were approved by the Institutional Animal Care and Use Committee at the Washington University School of Medicine (Assurance number A3381-01).

## Supporting information

S1 Table Antibody variable gene region sequence features for RRV antibodies. mRNA was isolated from clonal hybridoma cells, and cDNA was synthesized for Sanger automated DNA sequence analysis. The specific gene, allele, CDR length, and V-D and D-J junctions were determined through use of the IMGT program.
(PDF)

S2 Table Summary of neutralization of different RRV strains. The IC_50_, or concentration that gives a 50% reduction with accompanying 95% credible intervals, is listed along with R^2^, or percent of the variability explained by the regression fit, and E_max_, the estimated percentage maximum neutralization.
(PDF)

S3 Table Primers used for qRT-PCR viral RNA quantification in mice studies. One-step qRT-PCR was performed using RRV-specific forward and reverse primers in addition to a probe with 6FAM 5′ dye.
(PDF)

S1 Fig Neutralizing activity of RRV mAbs against RRV strain T48. Red circles represent percent neutralization relative to control at different antibody concentrations. Logistic curves are indicated by solid lines, and 95% credible intervals are indicated by dashed lines. A line at 100% neutralization highlights mAbs that completely neutralize. Multiple experiments were performed in triplicate, and the best fit curve is shown.
(PDF)

S2 Fig Neutralization profiles for five clinical isolate strains of RRV tested against four antibodies using a focus reduction neutralization test. RRV strains PW7 and SN11 were isolated from adult patients in 2009. RRV strain 2897601 (QML 2006) was isolated from an adult patient in 2006, and RRV strain O’Regan was isolated from an EP patient. The P7 and P14 isolates have been sequenced, and four mutations in the E2 protein were uncovered in the P7 strain: I76L, D132N, S182P, and R251K; for the P14 strain, there are two mutations in the E2 protein: I67L and R251K [15, 48, 49]. Red circles represent percent neutralization relative to control at different antibody concentrations. Logistic curves are indicated by solid lines, and 95% credible intervals are indicated by dashed lines. Multiple experiments were performed in triplicate, and the best fit curve is shown.
(PDF)

S3 Fig Binding of antibody to mutant residues relative to WT surface-expressed RRV proteins in alanine scanning mutagenesis experiments. A cutoff value of 10% (indicated by red dotted line) was used to determine mAb loss-of-binding at a residue, with the requirement that two other mAbs have binding of 50% or greater (indicated by the green dotted line). The orange colored graphs indicate mAbs meeting this requirement. The bar graphs represent the mean of two experiments, with the values from each individual experiment indicated by the white dots.
(PDF)

S1 Methods Logistic curve analysis used to calculate IC_50_ values for neutralization assays. (PDF)
