# Supplementary material for: Human monoclonal antibodies against Ross River virus target epitopes within the E2 protein and protect against disease
Source: PLoS Pathog. 2020 May 4;16(5):e1008517. doi: 10.1371/journal.ppat.1008517 (PMC7252634; doi:10.1371/journal.ppat.1008517)
Supplement: S1 Table — mRNA was isolated from clonal hybridoma cells, and cDNA was synthesized for Sanger automated DNA sequence analysis. The specific gene, allele, CDR length, and V-D and D-J junctions were determined through use of the IMGT program. (PDF) [file ppat.1008517.s001.pdf]

Table S1. Antibody variable gene region sequence features for RRV antibodies. mRNA was isolated from clonal hybridoma cells, and cDNA was synthesized for Sanger automated DNA sequence analysis. The specific gene, allele, CDR length, and V-D and D-J junctions were determined through use of the IMGT program.

Name	V-GENE and allele	J-GENE and allele	D-GENE and allele	CDR-IMGT lengths	AA JUNCTION
**RRV 6-HC**	*IGHV3-23*04 F,* or *IGHV3-23D*02 F*	*IGHJ5*01 F,* or *IGHJ5*02 F* (see comment)	*IGHD6-13*01 F*	8.8.11	CANLYISSWYLYW
**RRV 6-LC**	*IGKV3-15*01 F*	*IGKJ1*01 F*		6.3.11	CQQYNDWPPGGTF
**RRV 12-HC**	*IGHV4-31*03 F*	*IGHJ3*02 F*	*IGHD5-24*01 ORF*	10.7.15	CARVGGDGNNRDAFDIW
**RRV 12-LC**	*IGLV3-1*01 F*	*IGLJ3*02 F*		6.3.9	CQAWDSTTGVF
**RRV 19-HC**	*IGHV3-21*01 F*	*IGHJ6*02 F*	*IGHD2-15*01 F*	8.8.18	CARDDCSGSSCYYYYGMDVW
**RRV 19-LC**	*IGKV2-28*01 F,* or *IGKV2D-28*01 F*	*IGKJ3*01 F*		10.3.11	CMQALQTPRSFTF
**RRV 34-HC**	*IGHV3-7*01 F*	*IGHJ4*02 F*	*IGHD6-19*01 F*	8.8.13	CARDFDSSGWFPAYW
**RRV 34-LC**	*IGLV8-61*01 F*	*IGLJ3*02 F*		9.3.X	CLLYMISVLS#VF
**RRV 49-HC**	*IGHV4-61*01 F*	*IGHJ4*02 F*	*IGHD3-22*01 F*	10.7.14	CARGTRGYPQGYPDSW
**RRV 49-LC**	*IGLV3-10*01 F*	*IGLJ3*02 F*		6.3.10	CYSTDSSGTPLF
**RRV 86-HC**	*IGHV2-70*01 F*	*IGHJ4*02 F*	*IGHD6-13*01 F*	10.7.11	CTGGSSWYVPDYW
**RRV 86-LC**	*IGLV1-44*01 F*	*IGLJ2*01 F,* or *IGLJ3*01 F*		8.3.12	CAVWDNSLNGRVVF
**RRV 92-HC**	*IGHV3-15*01 F*	*IGHJ4*02 F*	*IGHD3-10*01 F*	8.10.19	CVNEAYYYGSGSYWEYYFDYW
**RRV 92-LC**	*IGLV3-19*01 F*	*IGLJ2*01 F,* or *IGLJ3*01 F*		6.3.10	CNSRDTSGNHLF

**RRV 130-HC**	*IGHV1-24*01 F*	*IGHJ2*01 F*	*IGHD3-10*01 F*	8.8.21	CTTGSYYHGWGSYSGRNWYFDLW
**RRV 130-LC**	*IGLV3-19*01 F*	*IGLJ2*01 F,* or *IGLJ3*01 F*		6.3.6	CNSRDSGF
**RRV 133-HC**	*IGHV3-30*03 F,* or *IGHV3-30*18 F* or *IGHV3-30-5*01 F*	*IGHJ6*02 F*	*IGHD3-3*02 F*	8.8.23	CARDQGLEVAFSQWSHYDYYGMDVW
**RRV 133-LC**	*IGLV2-14*01 F*	*IGLJ2*01 F,* or *IGLJ3*01 F*		9.3.11	CSSYTSISTSVLF
**RRV 135-HC**	*IGHV1-24*01 F*	*IGHJ4*02 F*	*IGHD3-10*01 F*	8.8.19	CATVAHYHGSDSYYNFYFDFW
**RRV 135-LC**	*IGLV3-19*01 F*	*IGLJ2*01 F,* or *IGLJ3*01 F*		6.3.12	CTSRDSSSSDLVIF
**RRV 136-HC**	*IGHV3-15*01 F*	*IGHJ5*02 F*	*IGHD2-8*01 F*	8.10.10	CMTESDMTFDPW
**RRV 136-LC**	*IGKV2-28*01 F,* or *IGKV2D-28*01 F*	*IGKJ3*01 F*		11.3.9	CMQALQTPYTF
**RRV 139-HC**	*IGHV3-23*04 F*	*IGHJ6*02 F*	*IGHD4-17*01 F*	8.8.16	CATATTVTYYYYYGMDVW
**RRV 139-LC**	*IGKV2-28*01 F,* or *IGKV2D-28*01 F*	*IGKJ3*01 F*		11.3.10	CMQALQPPRFTF
**RRV-191 HC**	*IGHV4-38-2*01 F*	*IGHJ6*02 F*	*IGHD2-21*02 F*	9.7.15	CVRERVGVTSYFGMDVW
**RRV-191 LC**	*IGKV-39*01 F* or *IGKV1D-39*01 F*	*IGKJ4*01 F*		6.8.8	CHQSYTGNTF
**RRV-196 HC**	*IGHV3-15*01 F*	*IGHJ1*01 F*	*IGHD3-3*01 F*	8.8.22	CTTISYYDFWSGHYMGREEYFQHW
**RRV-196 LC**	*IGLV3-19*01 F*	*IGLJ2*01 F* or *IGLJ3*01 F*		6.3.12	CNSRDSSGDPHVVF
**RRV-199 HC**	*IGHV3-33*01 F,* or *IGHV3-33*05* or *IGHV3-33*06 F*	*IGHJ6*202 F*	*IGHD6-19*01*		
**RRV-199 LC**	*IGLV2-8*01 F*			9.3.11	CSSYADNNNFVVF

		*IGLJ2*01 F,* or *IGLJ3*01 F*			
**RRV-200 HC**	*IGHV3-21*01 F,* or *IGHV3-21*02 F*	*IGHJ6*02 F*	*IGHD3-22*01 F*	8.8.28	CAREIVSYGDYGDSSGTSKYFYYYYGVDVW
**RRV-200 LC**	*IGKV1-12*01 F,* or *IGKV1-12*02,* or *IGKV1D-12*02 F*	*IGKJ3*01 F*		6.3.11	CQQANSFPPGFTF
**RRV-201 HC**	*IGHV3-33*01 F,* or *IGHV3-33*05 F,* or *IGHV3-33*06 F*	*IGHJ6*02*	*IGHD6-19*01 F*	8.8.18	CARDQGQWLVRPGWYGMDVW
**RRV-201 LC**	*IGLV2-8*01 F*	*IGLJ2*01 F,* or *IGLJ3*01 F*		9.3.11	CSSYADNNNFVVF
**RRV-205 HC**	*IGHV3-73*02 F*	*IGHJ6*02*	*IGHD5-12*01 F*	8.10.16	CLGNSGYDPEYFYGMDVW
**RRV-205 LC**	*IGLV6-57*01 F,* or *IGLV6-57*02 F*	*IGLJ2*01 F,* or *IGLJ3*01 F*		8.3.9	CQSYDRSNVVF
**RRV-207 HC**	*IGHV4-34*01 F*	*IGHJ4*02 F*	*IGHD6-13*01 F*	8.7.13	CARYVLGSSWPFDYW
**RRV-207 LC**	*IGKV1-39*01 F,* or *IGKV1D-39*01*	*IGKJ4*01 F*		6.3.9	CQQSSSTPLTF
**RRV-210 HC**	*IGHV3-11*01F*	*IGHJ5*02 F*	*IGHD1-26*01 F*	8.8.16	CARSVMGGTTMGEWFDPW
**RRV 210 LC**	*IGLV1-40*01 F*	*IGLJ2*01 F,* or *IGLJ3*01 F*		9.3.12	CQSYDSSLSGSVVF
**RRV-221 HC**	*IGHV1-18*01 F*	*IGHJ6*02 F*	*IGHD3-10*01 F*	8.8.22	CAREEGITLVRGTSNYYYYGMDVW
**RRV-221 LC**	*IGKV2-28*01 F,* or *IGKV2D-28*01 F*	*IGKJ2*01 F*		11.3.9	CMQALQTPHTF
