# Supplementary material for: Human monoclonal antibodies against Ross River virus target epitopes within the E2 protein and protect against disease
Source: PLoS Pathog. 2020 May 4;16(5):e1008517. doi: 10.1371/journal.ppat.1008517 (PMC7252634; doi:10.1371/journal.ppat.1008517)
Supplement: S2 Table — The IC50, or concentration that gives a 50% reduction with accompanying 95% credible intervals, is listed along with R2, or percent of the variability explained by the regression fit, and Emax, the estimated percentage maximum neutralization. (PDF) [file ppat.1008517.s002.pdf]

Table S2. Summary of neutralization of different RRV strains. The IC_{50}, or concentration that gives a 50% reduction with accompanying 95% credible intervals, is listed along with R^2, or percent of the variability explained by the regression fit, and E_{max}, the estimated percentage maximum neutralization.

RRV strain	MAb	Neutralization IC_{50} (ng/mL)	[95% credible interval]	R^2	E_{max}
T48	RRV-6	2,294	[509 – 12,228]	0.59	66
	RRV-12	63	[8.3 - > 2E5]	0.73	70
	RRV-19	16	[13 - 23]	0.92	99
	RRV-34	286	[16 - > 2E5]	0.48	61
	RRV-49	58	[26 – 128]	0.90	99
	RRV-86	174	[55 - 636]	0.72	65
	RRV-92	11	[2.7 - 57]	0.95	91
	RRV-130	5.7	[2.9 - 10]	0.78	101
	RRV-133	29	[21 - 41]	0.94	101
	RRV-135	10	[6.3 - 14]	0.88	93
	RRV-136	988	[157 - > 2E5]	0.73	67
	RRV-139	47	[14 - 135]	0.80	96
	RRV-191	334	[142 - 905]	0.79	96
	RRV-196	75	[23 - 205]	0.85	96
	RRV-199	6.7	[2.8 - 18]	0.86	95
	RRV-200	27	[18 - 42]	0.94	100
	RRV-201	49	[21 - 112]	0.86	93
	RRV-205	334	70 – 2,288]	0.56	70
	RRV-207	1,487	[901 – 2,429]	0.92	92
	RRV-210	13	[7.8 - 22]	0.87	100
	RRV-221	33	[6.8 - 217]	0.87	90
PW7	RRV-19	12	[8.2 - 16]	0.93	97
	RRV-92	18	[8.6 - 43]	0.81	81
	RRV-130	4.1	[1.3 - 8.4]	0.77	100

	RRV-135	12	[6.9 - 19]	0.85	94
SN11	RRV-19	10	[7 - 14]	0.92	96
	RRV-92	17	[11 - 34]	0.88	80
	RRV-130	9.0	[5.9 - 13]	0.9	99
	RRV-135	8.7	[5.9 - 13]	0.86	94
PW14	RRV-19	13	[6.9 - 28]	0.65	86
	RRV-92	70	[29 - 202]	0.86	74
	RRV-130	11	[8.5 - 14]	0.94	96
	RRV-135	14	[10 - 23]	0.89	92
2897601	RRV-19	12	[9.9 - 15]	0.95	95
	RRV-92	41	[16 - 147]	0.85	79
	RRV-130	13	[7.4 - 24]	0.84	98
	RRV-135	15	[8 - 38]	0.72	82
O’Regan	RRV-19	12	[9.2 - 14]	0.94	94
	RRV-92	105	[41 - 313]	0.79	81
	RRV-130	12	[9.5 - 16]	0.94	97
	RRV-135	17	[12 - 25]	0.94	96
