# Supplementary material for: Human monoclonal antibodies against Ross River virus target epitopes within the E2 protein and protect against disease
Source: PLoS Pathog. 2020 May 4;16(5):e1008517. doi: 10.1371/journal.ppat.1008517 (PMC7252634; doi:10.1371/journal.ppat.1008517)
Supplement: S3 Table — One-step qRT-PCR was performed using RRV-specific forward and reverse primers in addition to a probe with 6FAM 5′ dye. (PDF) [file ppat.1008517.s003.pdf]

Table S3. Primers used for qRT-PCR viral RNA quantification in mice studies.

One-step qRT-PCR was performed using RRV-specific forward and reverse primers in addition to a probe with 6FAM 5′ dye.

**Primer Name**	**Sequence**
RRV_For	GTGTTCTCCGGAGGTAAAGATAG
RRV_Rev	TCGCGGCAATAGATGACTAC
RRV_probe	5′6-FAM/ACCTGTTTA/ZEN/CCGCAATGGACACCA/3′IABkFQ
