# Supplementary material for: Human monoclonal antibodies against Ross River virus target epitopes within the E2 protein and protect against disease
Source: PLoS Pathog. 2020 May 4;16(5):e1008517. doi: 10.1371/journal.ppat.1008517 (PMC7252634; doi:10.1371/journal.ppat.1008517)
Supplement: S1 Fig — Red circles represent percent neutralization relative to control at different antibody concentrations. Logistic curves are indicated by solid lines, and 95% credible intervals are indicated by dashed lines. A line at 100% neutralization highlights mAbs that completely neutralize. Multiple experiments were performed in triplicate, and the best fit curve is shown. (PDF) [file ppat.1008517.s004.pdf]

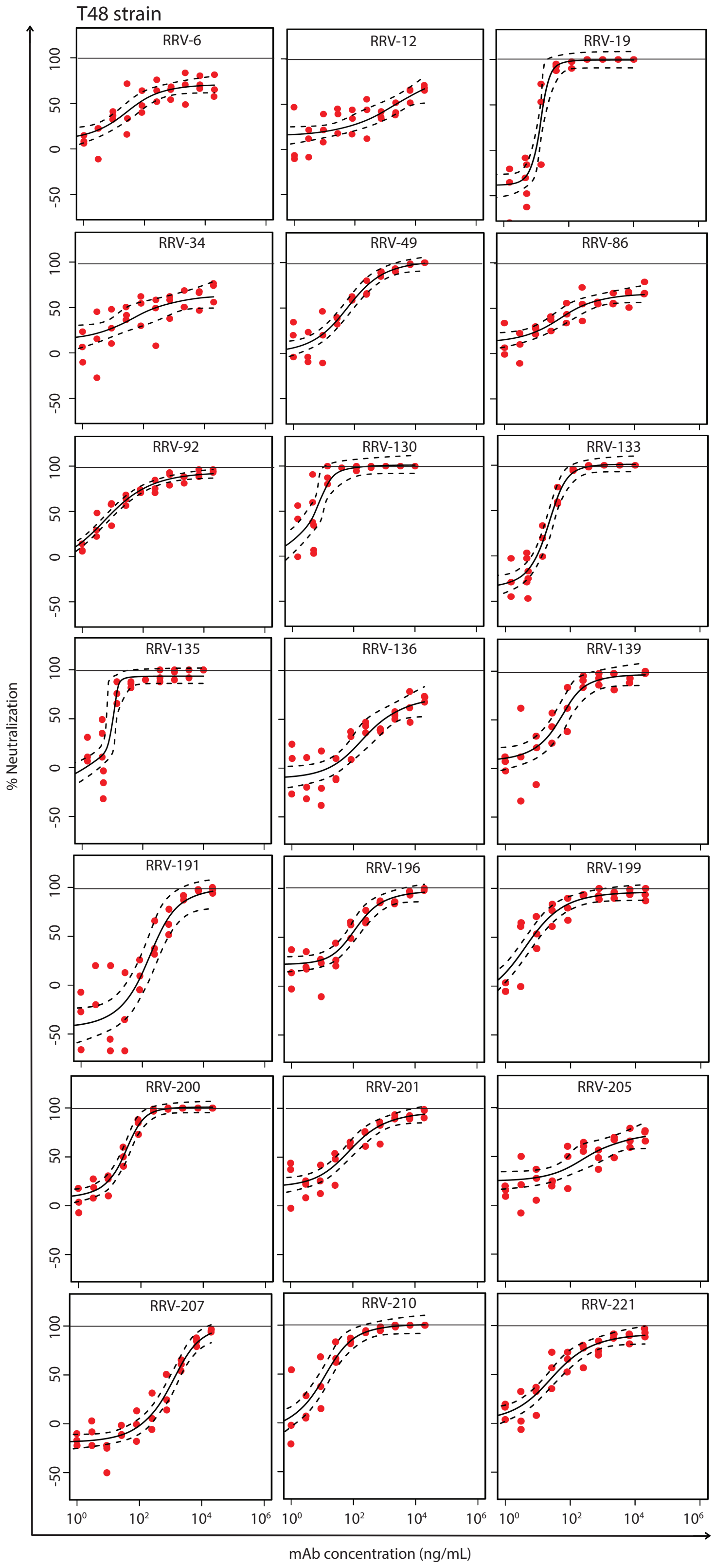

Figure S1. Neutralization activity of RRV mAbs against RRV strain T48. Red circles represent percent neutralization relative to control at different antibody concentrations. Logistic curves are indicated by solid lines, and 95% credible intervals are indicated by dashed lines. A line at 100% neutralization highlights mAbs that completely neutralize. Multiple experiments were performed in triplicate, and the best fit curve is shown.
