# Supplementary material for: Human monoclonal antibodies against Ross River virus target epitopes within the E2 protein and protect against disease
Source: PLoS Pathog. 2020 May 4;16(5):e1008517. doi: 10.1371/journal.ppat.1008517 (PMC7252634; doi:10.1371/journal.ppat.1008517)
Supplement: S2 Fig — RRV strains PW7 and SN11 were isolated from adult patients in 2009. RRV strain 2897601 (QML 2006) was isolated from an adult patient in 2006, and RRV strain O’Regan was isolated from an EP patient. The P7 and P14 isolates have been sequenced, and four mutations in the E2 protein were uncovered in the P7 strain: I76L, D132N, S182P, and R251K; for the P14 strain, there are two mutations in the E2 protein: I67L and R251K [15, 48, 49]. Red circles represent percent neutralization relative to control at different antibody concentrations. Logistic curves are indicated by solid lines, and 95% credible intervals are indicated by dashed lines. Multiple experiments were performed in triplicate, and the best fit curve is shown. (PDF) [file ppat.1008517.s005.pdf]

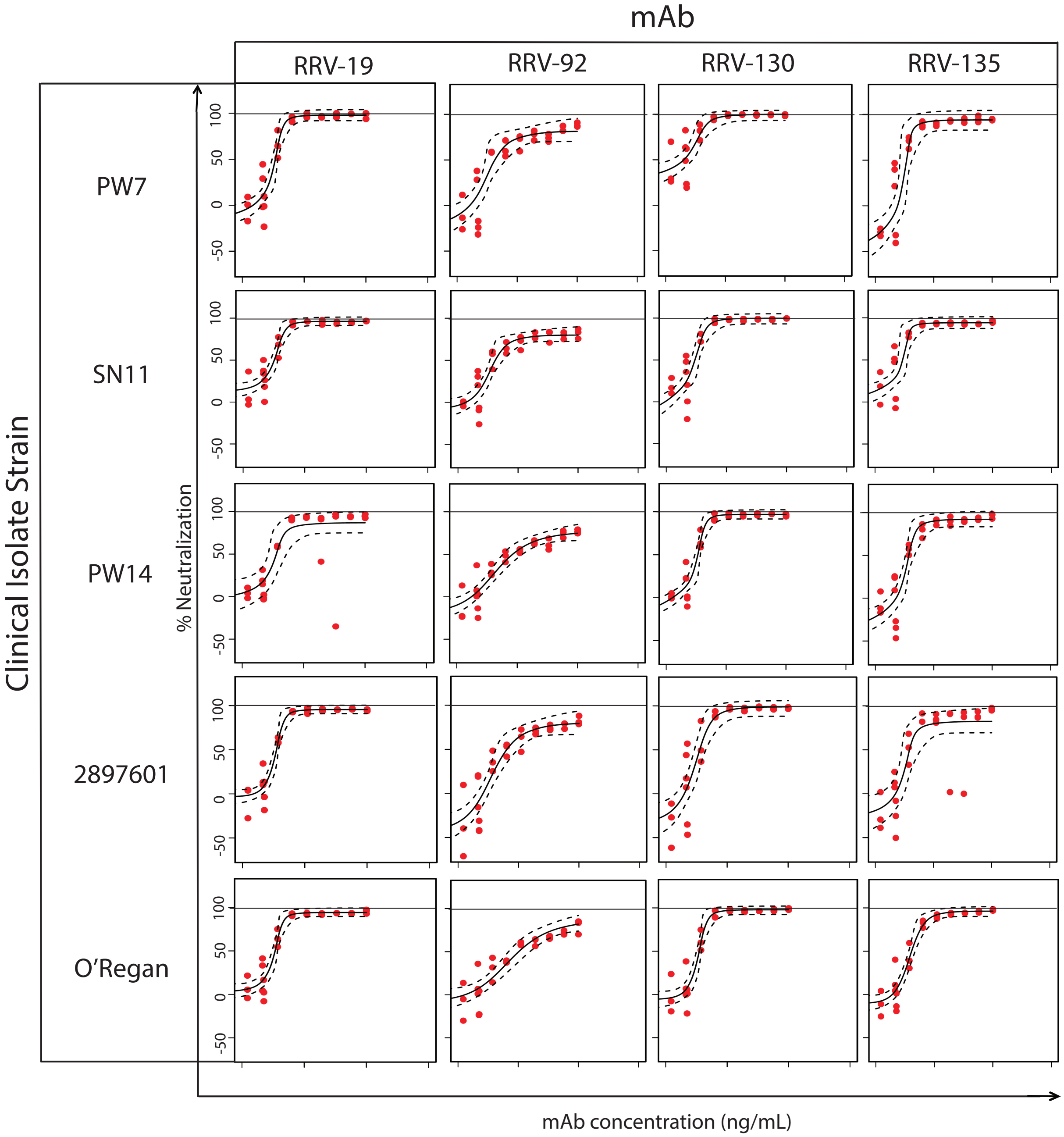

Figure S2. Neutralization profiles for five clinical isolate strains of RRV tested against four antibodies using a focus reduction neutralization test. RRV strains PW7 and SN11 were isolated from adult patients in 2009. RRV strain 2897601 (QML 2006) was isolated from an adult patient in 2006, and RRV strain O'Regan was isolated from an EP patient. The P7 and P14 isolates have been sequenced, and four mutations in the E2 protein were uncovered in the P7 strain: I76L, D132N, S182P, and R251K; for the P14 strain, there are two mutations in the E2 protein: I67L and R251K [15, and Aaskov et al., 1997, Liu et al., 2011 (below*)]. Red circles represent percent neutralization relative to control at different antibody concentrations. Logistic curves are indicated by solid lines, and 95% credible intervals are indicated by dashed lines. Multiple experiments were performed in triplicate, and the best fit curve is shown. *Additional references: Aaskov J, Williams L, Yu S. A candidate Ross River virus vaccine: preclinical evaluation. Vaccine. 1997;15: 1396–1404, and Liu WJ, Rourke MF, Holmes EC, Aaskov JG. Persistence of multiple genetic lineages within intrahost populations of Ross River virus. J Virol. 2011;85: 5674–5678.
