# Supplementary material for: Human monoclonal antibodies against Ross River virus target epitopes within the E2 protein and protect against disease
Source: PLoS Pathog. 2020 May 4;16(5):e1008517. doi: 10.1371/journal.ppat.1008517 (PMC7252634; doi:10.1371/journal.ppat.1008517)
Supplement: S3 Fig — A cutoff value of 10% (indicated by red dotted line) was used to determine mAb loss-of-binding at a residue, with the requirement that two other mAbs have binding of 50% or greater (indicated by the green dotted line). The orange colored graphs indicate mAbs meeting this requirement. The bar graphs represent the mean of two experiments, with the values from each individual experiment indicated by the white dots. (PDF) [file ppat.1008517.s006.pdf]

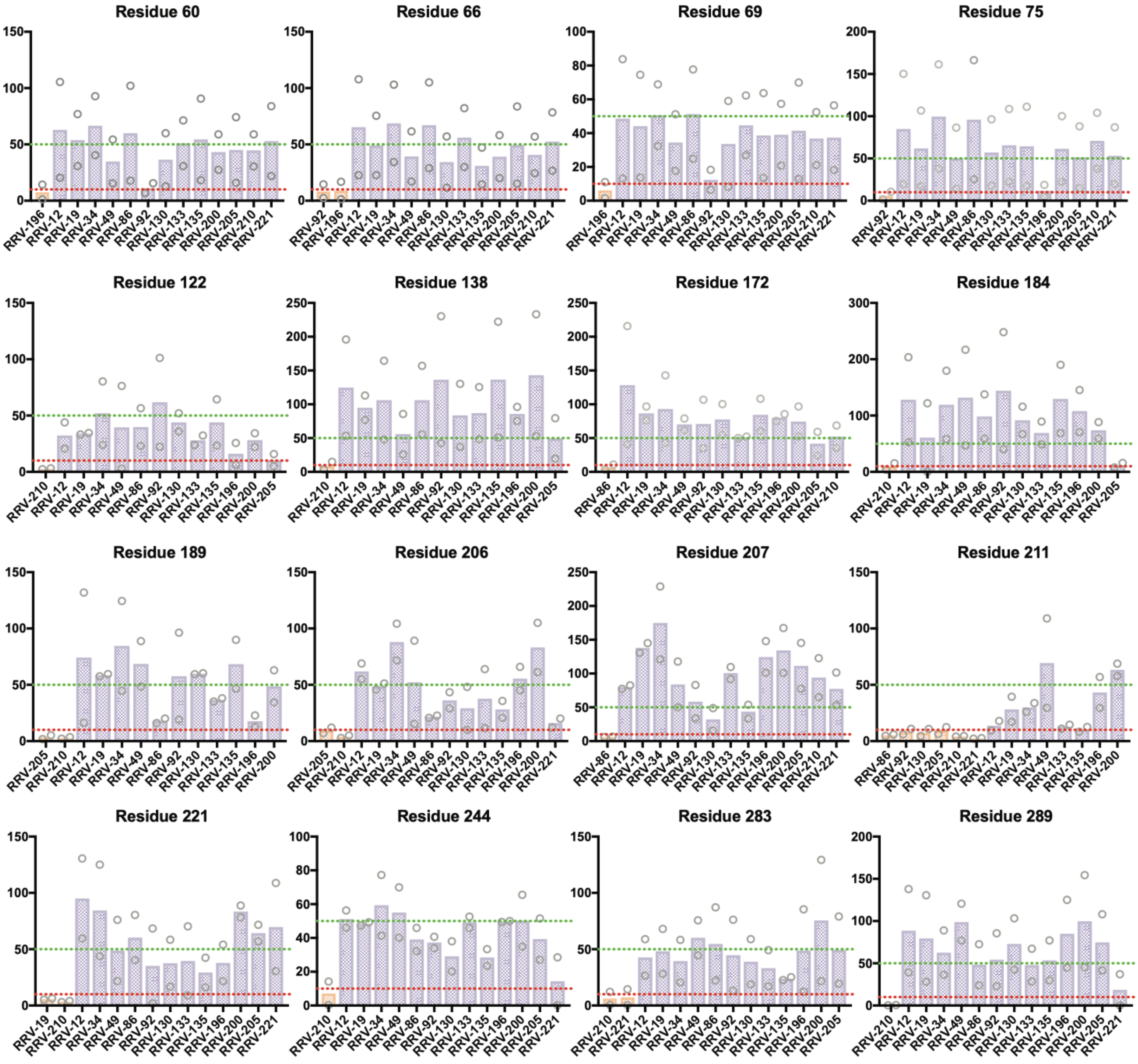

Figure S3. Graphs showing percent binding of antibody to mutant residues relative to WT surface-expressed RRV proteins in alanine scanning mutagenesis. A cutoff value of 10% (indicated by red dotted line) was used to determine mAb loss-of-binding at a residue, with the requirement that two other mAbs have binding of 50% or greater (indicated by the green dotted line). The orange colored graphs indicate mAbs meeting this requirement. The bar graphs represent the mean of two experiments, with the values from each individual experiment indicated by the white dots.
