# Supplementary material for: Human monoclonal antibodies against Ross River virus target epitopes within the E2 protein and protect against disease
Source: PLoS Pathog. 2020 May 4;16(5):e1008517. doi: 10.1371/journal.ppat.1008517 (PMC7252634; doi:10.1371/journal.ppat.1008517)
Supplement: S1 Methods — (PDF) [file ppat.1008517.s007.pdf]

SUPPLEMENTAL METHODS

Logistic curve analysis used to calculate IC_{50} values for neutralization assays

The logistic growth curve we considered for concentration x includes five parameters β_1, β_2, β_3, β_4, and β_5. The function follows the following form:

$$f(x) = \beta_1 + \frac{\beta_2 - \beta_1}{1 + g_x \exp\left(\frac{\beta_3 - x}{\beta_4}\right) + (1 - g_x)\exp\left(\frac{\beta_3 - x}{\beta_5}\right)}$$

where

$$g_x = \frac{1}{1 + \exp\left(-\overline{C}_g(\beta_3 - x)\right)}$$

is a logistic weighting function varying smoothly between 0 and 1, centered about the 50% reduction point. If β_4 and β_5 are the same sign, then the mean curvature of g was determined by the reciprocal of the mean

$$\overline{C}_g = \frac{2}{|\beta_4 + \beta_5|}$$

β_1 is the lower asymptote; β_2 is the upper asymptote; β_3 is inflection point of the function which corresponds to a 50% reduction point; β_4 and β_5 controls the relative steepness or shallowness of the logistic curve (note that this parameterization does not assume symmetry).
